# Brief comment on “Mitral valve repair and replacement in infectious endocarditis: a systematic review and meta-analysis of clinical outcome”

**DOI:** 10.1186/s43044-025-00610-w

**Published:** 2025-01-30

**Authors:** Harvey James Moldon, Armia Ahmadi-Hadad

**Affiliations:** University of Naples “Federico II”, Naples, Italy

The advantages and disadvantages of mitral valve repair and replacement in the setting of infective endocarditis represent a valuable clinical question, and certainly merit thorough investigation. For this reason, we read Nuthalapti et al.’s work “Mitral valve repair and replacement in infectious endocarditis: a systematic review and meta-analysis of clinical outcome” [1] with great interest.

We noticed that data extracted from Al-Zubaidi et al. [2] include non-infective causes of endocarditis, and that data extracted from Navia et al. [3] include aortic valve replacement procedures. This calls into question the utility of this data as a basis for clinical decision-making in the context of mitral valve infective endocarditis, seeing as they are derived from patients with non-mitral valve endocarditis and patients with non-infective (e.g. rheumatic) mitral valve endocarditis.

Furthermore, we noticed that some of the studies included in the analysis do not appear to be retrospective studies, and instead are themselves systematic reviews and/or meta-analyses of secondary data (namely Feringa et al. [4] and Harky et al. [5]). This would likely lead to the duplication of large parts of the dataset, thus greatly exaggerating any existing patterns present within the collected data in any subsequent statistical analyses.

We were greatly interested in reading the large study by James et al. from 2005; however, we were unable to locate a citation for it.

We hope that by raising these points, we might help steer the discourse surrounding mitral valve repair and replacement in the direction of improved precision and clinical relevance, with the ultimate aim of a providing a solid foundation for evidence-based guidelines.

## Data Availability

No datasets were generated or analysed during the current study.

## References

[1] Nuthalapati U, Bathinapattla MR, Cardoso RP, Jesi NJ, Singh K, Moradi I, Gostomczyk K, Afzal M, Omer MB, Mian ZR, Patel S, Sachdeva P, Malik MN, Abbas M, Singh J, Shafique MA (2024) Mitral valve repair and replacement in infectious endocarditis: a systematic review and meta-analysis of clinical outcome. Egypt Heart J 76:134 39365370 10.1186/s43044-024-00564-5 PMC11452577

[2] Al-Zubaidi F, Pufulete M, Sinha S, Kendall S, Moorjani N, Caputo M, Angelini GD, Vohra HA (2023) Mitral repair versus replacement: 20-year outcome trends in the UK (2000–2019). Interdisciplinary CardioVascular and Thoracic Surgery 36(6):ivad086 37208195 10.1093/icvts/ivad086 PMC10250075

[3] Navia JL, Elgharably H, Hakim AH, Witten JC, Haupt MJ, Germano E, Houghtaling PL, Bakaeen FG, Pettersson GB, Lytle BW, Roselli EE, Gillinov AM, Svensson LG (2019) Long-term Outcomes of Surgery for Invasive Valvular Endocarditis Involving the Aortomitral Fibrosa. Ann Thorac Surg 108:1314–1323 31254508 10.1016/j.athoracsur.2019.04.119

[4] Feringa HHH, Shaw LJ, Poldermans D, Hoeks S, Van Der Wall EE, Dion RAE, Bax JJ (2007) Mitral Valve Repair and Replacement in Endocarditis: A Systematic Review of Literature. Ann Thorac Surg 83:564–570 17257988 10.1016/j.athoracsur.2006.09.023

[5] Harky A, Hof A, Garner M, Froghi S, Bashir M (2018) Mitral valve repair or replacement in native valve endocarditis? Systematic review and meta-analysis. J Card Surg 33:364–371 29926515 10.1111/jocs.13728

